# Applications of Hemp Polymers and Extracts in Food, Textile and Packaging: A Review

**DOI:** 10.3390/polym14204274

**Published:** 2022-10-12

**Authors:** Khwanchat Promhuad, Atcharawan Srisa, Horman San, Yeyen Laorenza, Phanwipa Wongphan, Janenutch Sodsai, Kittichai Tansin, Phannaphat Phromphen, Nawarat Chartvivatpornchai, Phurit Ngoenchai, Nathdanai Harnkarnsujarit

**Affiliations:** 1 Department of Packaging and Materials Technology, Faculty of Agro-Industry, Kasetsart University, 50 Ngam Wong Wan Rd., Latyao, Chatuchak, Bangkok 10900, Thailand; 2 Department of Textile Science, Faculty of Agro-Industry, Kasetsart University, 50 Ngam Wong Wan Rd., Latyao, Chatuchak, Bangkok 10900, Thailand; 3 Department of Product Development, Faculty of Agro-Industry, Kasetsart University, 50 Ngam Wong Wan Rd., Latyao, Chatuchak, Bangkok 10900, Thailand; 4 Center for Advanced Studies for Agriculture and Food, Kasetsart University, 50 Ngam Wong Wan Rd., Latyao, Chatuchak, Bangkok 10900, Thailand

**Keywords:** hemp (*Cannabis sativa* Linn), hemp fiber, hemp seed oil, packaging

## Abstract

Hemp (*Cannabis sativa* Linn.) is a high-yielding annual crop farmed for its stalk fiber and oil-producing seeds. This specialized crop is currently experiencing a revival in production. Hemp fiber contains pectin, hemicellulose and lignin with superior strength, while hemp seed oil contains unsaturated triglycerides with well-established nutritional and physiological properties. Therefore, focus on the utilization of hemp in various industries is increasing globally. This study reviewed recent applications of hemp components, including fiber and extract, in food, textile and packaging applications. Hemp fibers mainly consisting of cellulose derivatives have superior strength to be used as reinforcements in thermoplastic packaging and paper. Combined physical and chemical modifications of hemp fibers improved mechanical and barrier properties of composite materials. Physically and chemically processed hemp extracts have been used in food and non-food applications. Functional foods containing hemp oils deliver nutrients by their unsaturated lipids. High-quality hemp fiber with several fiber modifications has been applied in garments. Innovative applications of hemp components and by-products are increasing, thereby facilitating utilization of green sustainable biomaterials.

## 1. Introduction

Hemp has a long history of use in human civilization dating back to 8000 BC. It is one of the world’s oldest cultivated plants, having evolved from wild cannabis native to Central Asia [1] and then spread to Europe, Africa and South America where hemp seeds and oil were consumed. In China, hemp has been cultivated for over 6000 years. Early in the Christian era, hemp was cultivated in Mediterranean countries and spread throughout Europe during the Middle Ages. Hemp was first planted in Chile in the 1500s and in North America a century later. It has long been used for textiles as well as a nutritional source for food, cosmetics and oriental medicine [2,3]. Hemp is a dioecious, short-day photoperiod-sensitive annual crop capable of producing cannabinoids, fatty acids and other nutritional products for biomedical purposes [4]. Monoecious varieties of industrial hemp are more prevalent by plant breeding, particularly those cultivated in Europe. Due to its antioxidant and antimicrobial properties, it is also used as an active ingredient in commercial insect repellents and biopesticides [5,6]. Hemp has the highest Young’s modulus values of any natural fiber and is the strongest cellulose fibers. High aspect ratios (length/width) are another quality that makes hemp fibers ideal for use as reinforcement in composite materials. With better farming practices, hemp has potential for significantly higher fiber outputs and reduced costs [7]. Hemp seed oil contains vital fatty acids with health advantages such as lowering cholesterol, easing dermatitis and reducing high blood pressure and has recently experienced a resurgence in popularity [8]. Conversion of the hemp seed oil into polymeric materials is an economic, eco-friendly alternative solution for its disposal [9].

Most product classifications addressed here are based on the amounts of tetrahydrocannabinol (THC) and cannabidiol (CBD) present in the products. THC is a psychoactive chemical with limited medicinal applications, whereas CBD is non-psychotropic and has a greater number of medical uses. Increasing THC content is linked to a higher risk of abuse, necessitating tougher controls. However, the ban on industrial hemp has now been overturned and its economic and environmental advantages have been recognized. The US, the EU and other nations have recently legalized the growth of industrial hemp and many other nations have reintroduced hemp farming with low THC levels, producing a renaissance in hemp production [10]. Parties involved in both upstream and downstream production include healthcare experts, hospitals, consumers or patients and regulatory bodies. Traditional medicine practitioners are powerful stakeholders in Thailand, using long-established traditional cannabis formulations.

Drivers of sustainability culture are now focusing on opportunities to capitalize on the future of packaging. A growing number of countries have banned non-biodegradable and petroleum-based plastics for certain applications and bioplastics have become the natural replacement choice. Escalated researches are being carried out in the food and packaging industries for development of materials based on bioplastic of renewable origin [11,12].

## 2. Hemp Polymeric and Extract Components

**Figure 1:**
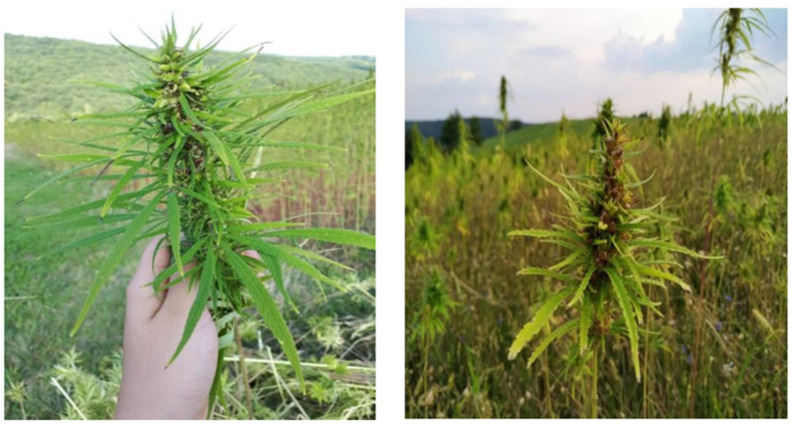
Appearance of hemp (*Cannabis sativa* Linn) [13].

**Figure 2:**
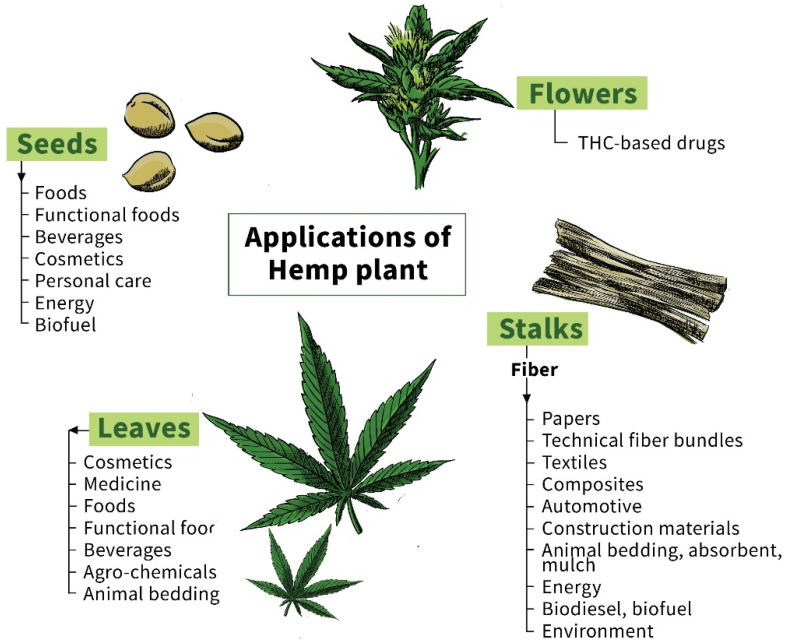
Applications of hemp plant components as seeds, flowers, leaves and stalks.

Hemp (Figure 1) is one of the world’s oldest bast plants and has long been used as a vital raw material in many civilizations (Figure 2). The most common reason for hemp cultivation is to isolate fibers from its stem bark to make ropes, textiles and paper. Other useful hemp materials include the seed, which can be used to make oil, and cannabinoids for medical, spiritual and recreational purposes. Plant maturity, separation processes, soil type and climatic conditions during growth impact hemp fiber quality as well as its chemical composition.

Hemp fibers are composed of cellulose, hemicellulose, lignin and pectin. Bast fibers have higher cellulose content and are stronger than hurd fibers which have higher lignin content and are unsuitable as composite material reinforcement. The highly oriented crystalline structure of cellulose produces rigid fibers with robust tensile qualities that are vulnerable to the production of kink bands under compression and tension, adversely affecting fiber strength.

## 3. Hemp Polymeric Fiber and Nanofiber

The main components of hemp fiber (Table 1) are crystalline cellulose, accounting for 55–72% of fiber mass, hemicellulose (8–19%), lignin (2–5%) and lesser amounts of waxy compounds (Thomsen et al. 2000). The fibers can be modified to enhance interfacial bonding. Addition of chemical coupling agents and compatibilizers to the polymer matrix improves reactivity and wetting of the reinforcing fibers. Treatments on hemp fiber can be biological, physical or chemical to accomplish one or more of the following goals: (1) removal of unwanted fiber components; (2) roughen the fiber surface; (3) separate individual fibers from their fiber bundles; (4) change the chemical makeup of the fiber surface and (5) reduce the hydrophilicity.

The process can continue to achieve nano-scale fiber by mechanical processes such as high-pressure homogenization, microfluidisation, grinding, refining, high-intensity ultra-sonication, cryo-crushing [25,26]. A nanocellulose fiber has diameter or width in range of 1–100 nm. Tyagi et al. (2021) [25] observed that hemp-based nanofiber was more fibrillated with smaller fiber diameter (45–98 nm) compared to hardwood-based nanofiber (72–136 nm). In particular, fibrillated hemp via hydrothermal process had the smallest fiber diameter (45 nm). This is because the hemicellulose content in hemp is higher than hardwood. Furthermore, the higher amount of hemicellulose, a higher negative surface charge on the fiber would achieve to facilitate the fibrillation because of swellability.

### 3.1. Preparation and Composition of Hemp Fiber

#### 3.1.1. Biological Retting

To separate bast fibers from their fiber bundles, woody core and epidermis, plant stems must be subjected to controlled degradation or retting. During the retting process, bacteria and fungi release enzymes that break down pectic and hemicellulose substances in the central lamella between the various fiber cells. Bast fibers become soft and clean as the strands separate from the woody core. Retting time is critical as over-retting can weaken fibers and increase fiber mass losses during processing, while under-retting can result in incomplete fiber separation. Retting can be done as dew retting when plant stems are left in the field to partially deteriorate or as water retting when plant stems are submerged in water. Although water retting produces consistent, high quality fabric, its application is limited due to issues with environmental degradation. Dew retting is a significantly slower process that can only be carried out in areas where enough dew is discharged at night [27].

Liu et al. (2016) [28] investigated the effect of duration of enzymatic retting using endo-polygalacturonase from *Emericella nidulans* produced via fermentation, which was then applied to hemp fiber. They found fewer impurities, greater fiber separation, lower parenchyma cells, and increased loosened bast fiber after increasing enzymatic retting duration up to 300 min. It also affected the porosity of hemp fiber composite. Enzymatic retting removed the parenchyma cells on the fiber surface which improved coherence between the hemp fiber and composited matrix, thus the porosity was reduced.

#### 3.1.2. Steam Explosion and Plasma Treatment

Steam explosion is an efficient and low-energy method of fiber separation as an alternative to environmentally harmful fiber separation methods like water retting. Compared to conventional retting techniques, steam explosion takes less time and is easier to control [29]. Semi-retted bast fiber is separated from the woody core and impregnated with a weak solution of sodium hydroxide under vacuum. The fibers are drained before loading into a steam reactor and steamed for 90 s at 2000 °C (1.5 MPa) [29]. The quick release of pressure from the steam reactor causes explosive decompression. The fiber bundles are blown apart and separated at the center lamella as water in the fiber rapidly vaporizes and expands in volume. During the steam treatment, pectin, hemicelluloses and lignin are partially broken down and solubilized in the sodium hydroxide solution, resulting in increased fiber separation.

The chemical and physical nature of fiber surface layers can be modified via plasma treatment. Physical changes take place as a result of the sputtering effect that roughens the fiber surface, expands the fiber contact area and increases friction between the fiber and the polymer matrix. Depending on the kind and composition of plasma gas utilized, chemical alterations can promote attachment of active polar groups on the fiber surface which lowers surface energy and encourages chemical bonding between the fibers and the polymer system. Fiber surface energy, surface cross-linking and of reactive free radical generation are also increased after plasma treatment.

#### 3.1.3. Alkaline Treatment

**Figure 3:**
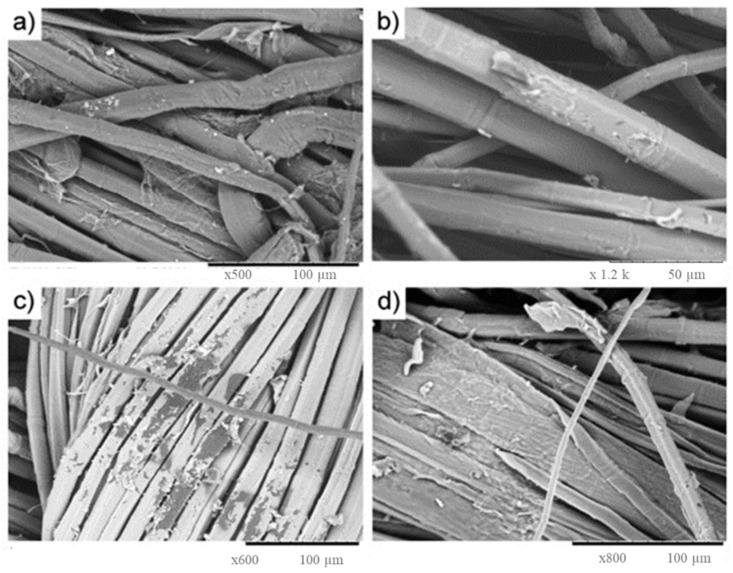
SEM surface images (**a**) untreated, (**b**) 1% silane-treated, (**c**) 5% silane-treated and (**d**) 20% silane-treated hemp fibers (Reproduced with permission from Sepe et al. (2018) [31]).

Treatment of hemp fiber with NaOH is frequently used to change the cellulose molecular structure. When alkali treatment is carried out, particularly at a high temperature, lignin, pectin and hemicellulose in the fiber wall are selectively degraded, while the cellulose components are largely unaffected. Natural fiber-reinforced composites are stronger as a result of the elimination of these cementing substances. Alkali treatments can easily remove pectin and hemicelluloses but lignin in hemp bast fiber are more difficult to eliminate. Lignins have strong carbon-carbon bonds and aromatic groups that are resistant to chemical attack and prevent degradation and fragmentation [30]. Sepe et al. (2018) [31] presented SEM images (Figure 3) which indicated that alkali treatment effectively eliminated both lignin and hemicellulose from the surface of hemp fibers; however, alkali treatment contributes to fibrillation (loss of fibril from the fiber) that decreases the mechanical properties of the composites. Kabir et al. (2013) [32] found that higher alkaline concentration (4–10% NaOH) efficiently removed hemicellulose and lignin, while silane treatment was ineffective. Islam et al. (2011) [33] treated hemp fibers with 5% NaOH and 2% Na_2_SO_3_ (120 °C and 60 min) and concluded that alkali treatment effectively (i) eliminated lignin, (ii) separated the fibers from fibrous bundles, (iii) exposed the O-H groups in cellulose, (iv) cleaned the surface of the hemp fibers and (v) improved chain packaging and increased crystallinity in cellulose components which improved thermal stability.

#### 3.1.4. Acetylation Treatment

Acetylation treatment employs use of acetic anhydride to increase fiber dispersion within the polymer matrix and reduce the hydrophilic properties of cellulose fibers [34]. Acetic anhydride is a compatibilizer that reduces the surface energy of the fibers, rendering them nonpolar and improves its compatibility with the polymer [35]. This is accomplished through interaction between the hydrophilic hydroxyl groups (-OH) of hemp fiber and acetyl groups (-CH, -CO) of acetic anhydride. The -OH fiber groups become unreactive and can no longer form bonds with other -OH groups, water or other chemicals once they have linked with the acetyl groups. Hajiha et al. (2014) [36] also showed that combination acetylation and alkaline treatments resulted in high rate of removal of hemicellulose and lignin from hemp fiber (indicated by FTIR) and decreased hydrophilicity of the fiber leading to the low moisture content.

#### 3.1.5. Silane Treatment

The composition of silane coupling agents enables the formation of a siloxane bridge between the surface of cellulose fiber and resin. In the presence of moisture, hydrolysable alkoxy groups in these agents form silanols that that react with the hydroxyl groups of fibers at one end and the functional matrix groups at the other. This core activity provides molecular continuity across the interfacial region of the composite and improves fiber-matrix adhesion. Silane-treated fiber composites have higher tensile strength than alkali-treated fiber composites [37]. Sepe et al. (2018) [31] used vacuum infusion with a trimethoxysilane coupling agent to produce hemp fiber reinforced epoxy composites that had improved mechanical strength. Chemical treatment with 1% silane (optimal concentration) gave 15% and 10% higher tensile modulus than hemp fiber treated with 5% NaOH and untreated hemp fiber composites, respectively. Kabir et al. (2013) [32] also indicated that silane treatments enhanced fiber coupling with silane molecules, particularly on fiber surfaces, and increased the weight by filling spaces between the microfibrils.

#### 3.1.6. Maleic Anhydride Treatment

Maleic anhydride (MA) is used to improve interfacial bonding and mechanical properties in composites by modifying both the fiber surfaces and the thermoplastic matrices. Maleic anhydride-grafted polypropylene (MAPP) is a compatibilizer and coupling agent made up of long polymer chains that have been grafted with an MA functional group. MAPP acts as a bridge between the nonpolar PP matrix and polar fibers by chemically bonding with cellulose fibers via MA groups and bonding to the matrix via polymer chain entanglement. MA functional groups interact strongly with reactive OH groups on the surface of cellulose and lignin through covalent and hydrogen bonding. MAPP polymer chains then combine with the unreactive PP matrix via chain entanglement [38]. These entanglements act as physical cross-links, providing mechanical integrity up to and above the glass transition temperature of the matrix. The MAPP polymer chain length is an important factor in determining the level of chain entanglement that the coupling agent can achieve. Short MAPP polymer chains reduce the chance of entanglement between the coupling agent and matrix chains because they can easily slide past each other. Entanglement can occur when the MAPP polymer chains are longer, but viscosity of the coupling agent also increases, resulting in poor fiber wetting. If the MAPP chains are extremely long, they may entangle with PP molecules, making it more difficult for the MA groups in MAPP to migrate to OH groups on the fiber surface.

## 4. Hemp Polymeric Composites in Packaging Applications

### 4.1. Package Forming Technology

#### 4.1.1. Melt Mixing

Melt mixing using a radial flow (turbulent) mixer is a common method for combining thermoplastic polymers with short reinforcing fibers. The thermoplastic polymer is first heated to its melting point before the hemp fibers are added to the mix. The composite mixture can then be rolled into a sheet or formed into other shapes. Parameters such as mixing duration, rotor speed and melt-chamber temperature influence the composite properties. Gironès et al. (2012) [39] developed reinforced thermoplastic starch and hemp fiber composites by melt processing.

#### 4.1.2. Solution Casting

**Figure 4:**
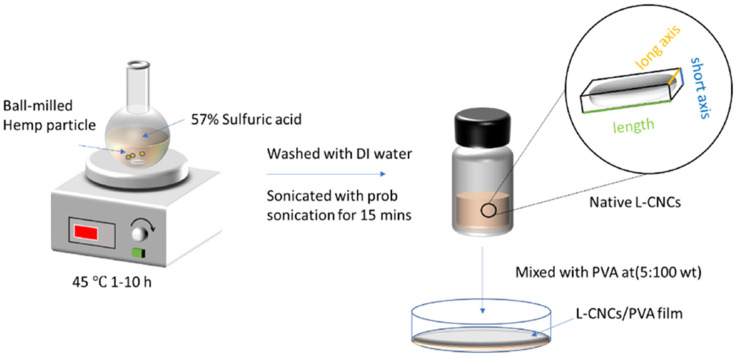
Schematic representation of solution casting (Reproduced with permission from Zhang et al. 2020 [42]).

Solution casting is an alternative to physical mixing methods [40] such as melt mixing, extrusion and injection molding, in which the polymer is melted during fiber compounding. The polymer is first dissolved in a suitable solvent to which the fiber is then added and the polymer is precipitated from the solvent in a vacuum oven. Solution casting uses low temperatures and shear stresses, thereby preventing fiber damage that occurs during blending fibers and thermoplastics via melt mixing and extrusion compounding. Luzi et al. (2016) [41] prepared PLA and PLA PBS-based cellulose nanocrystal films by solvent casting, while Zhang et al. (2020) and Zhang et al. (2021) [42,43] prepared PVA and hemp fiber films by solvent casting following the method shown in Figure 4.

#### 4.1.3. Extrusion Compounding

Extrusion is a highly efficient method of combining natural fibers and thermoplastic polymers [44,45,46,47]. A thermoplastic polymer (pellet or powder form) and short hemp fibers are combined in a heated extrusion barrel by single or twin screws depending on the type of extruder. Short fiber-reinforced thermoplastic composites are frequently extruded using a twin-screw extruder before injection molding, due to the excellent fiber distribution achieved within the polymer matrix. To achieve uniform fiber distribution, extruder processing variables such as barrel length, temperature profile, screw configuration and screw speed are optimized. Beckermann et al. (2007) [48] used a twin-screw extruder and injection molding machine to produce composite materials using either treated or untreated fiber, polypropylene and a maleic anhydride-modified polypropylene (MAPP) coupling agent. Terzopoulou et al. (2016) [49] produced composite materials comprising poly(butylene succinate) and hemp as fillers (15, 30, 50, 60 and 70%) by melt mixing in a twin screw extruder. They found that tensile and impact strength depended on the type and amount of hemp fillers that acted as nucleating agents and increased the degradation rate in enzymatic hydrolysis and soil burial.

#### 4.1.4. Injection Molding

Injection molding is most widely used to produce molded parts from thermoplastic and reinforced thermoplastic materials, as it is one of the few manufacturing processes capable of producing net-shape composite parts in high volumes and at high production rates. Short hemp fiber-reinforced composites can be processed into complex-shaped components using standard thermoplastic injection molding equipment. Composite materials for injection molding applications must be capable of fluid-like flow during processing and typically consist of short fibers with a low fiber fraction. Low fiber content can result in insufficient reinforcement, while a high fiber content can result in poor molding and reduced composite mechanical properties. Injection-molded melts are typically performed by extrusion compounding composite pellets in a heated barrel to deliver a homogeneous melt to the machine nozzle for injection into a closed mold. Injection molding causes reduced mechanical friction on the composite melt compared to other mixing processes such as extrusion and melt mixing with less fiber damage. The mold unit has a fixed and movable section that encloses a shaped cavity into which the composite is injected and cooled to determine the final shape of the molded part. Fiber and polypropylene composites were first developed by Yan et al. (2013) [50]. An injection molding machine is used to shape the grains of the composite mixture into specimens for mechanical tests. Pappu et al. (2019) [7] used melt processing and injection molding procedures to create hybrid fiber reinforced biodegradable composites that combined sisal and hemp fiber with polylactic acid.

#### 4.1.5. Compression Molding

Compression molding is a common manufacturing technique for large, relatively simple composite parts with good mechanical properties. Compression molding involves hot pressing of randomly oriented or aligned fiber mats with a thermoplastic material, either chopped or in continuous form. The compression molding process begins by placing a stack of alternating fiber mats and thermoplastic sheets in the bottom half of a preheated mold cavity. The top half of the mold is then lowered at a constant rate until the desired processing pressure is reached, causing the polymeric matrix to melt and the composite to consolidate. After pressing, the composite is cooled and removed from the mold. Dhakal et al. (2012) [51] studied hemp fiber/unsaturated polyester composite laminates using a compression molding process, while Wretfors et al. (2009) [52] examined the potential of adding industrial hemp fibers to wheat gluten polymers by this process. Dayo et al. (2017), Dayo et al. (2018) and Dayo et al. (2019) [53,54,55] fabricated compression molded composites of polybenzoxazine incorporating hemp fibers.

### 4.2. Applications of Hemp Fiber

#### 4.2.1. Paper

Hemp has been used to make paper for centuries. Hemp fiber was initially used in ancient China as paper scrolls [56]. Hemp paper outperforms tree-based paper in terms of decomposition resistance and strength, especially when wet with improved yellowing resistance (Table 2). The high-quality physical pulp properties and tensile strength make hemp an ideal non-wood-based raw material for making specialty paper. Barbash et al. (2022) [57] developed hemp paper from fiber with density of up to 1.56 g/cm^3^, tensile strength of up to 66.7 MPa and transparency of up to 87.3%, while Cetin et al. (2022) [58] developed Turkish hemp-based paper coated with PLA film to improve barrier properties. These researchers indicated that hot pressing improved interface adhesion between the two layers, improving barrier (by around 50%) and mechanical properties and thermal stability (no shrinkage of paper over 100 °C to 200 °C). Amode and Jeetah (2021) [59] produced 100% Mauritian hemp paper and demonstrated the best pulping method (12% NaOH at 90 °C for 90 min), giving apparent density of 141.54 kg/m^3^ with water absorbency time as 1.436 s, while mechanical properties observed were burst strength of 0.323 kPa m^2^/g, tensile strength of 10.97 Nm/g, abrasion resistance of 37.5 cycles before rupture and crease recovery angle of 34.8°. Addition of tapioca starch (10–40%) efficiently improved tensile strength, burst strength, abrasion resistance, apparent density and resistance to water absorption [59]. Kirilovs et al. (2015) [60] developed hemp fibers/shives mix boards that can be utilized as insulating materials. Heat transfer was reduced due to their porous structure and low density. The physical and structural characteristics of materials influenced the heat insulation capabilities. Inexpensive particle board was made from chopped hemp stem and tested for heat conductivity. The samples were made with three different types of adhesives and varying thicknesses. Jianyong and Jianchun (2015) [61] improved properties of paper made from hemp root. It has been discovered that beating intensity and weight have significant impact on hemp paper properties such as air permeability, pore diameter distribution, oil/air penetration, and oil/air filtering effectiveness. Hemp papers have an oil filtration effectiveness of 99.7–99.975% for particles of 0.33 mm and an air filtration performance of 99.942–100% for NaCl aerosol particles 0.26 mm. Nabels-Sneiders et al. (2022) [62] studied porous cast hemp papers which were coated with melt of polyhydroxyalkanoate (PHA), PLA, polybutylene succinate (PBS), and polybutylene succinate adipate (PBSA). The composite was compressed under three different loads: 0.5 MT, 1.5 MT, and 3.0 MT. Elastic modulus and tensile strength were improved up to two-fold with an increase in compression pressure. Storage modulus of the laminates were impacted by the polymer’s transition into a viscoelastic state. Baptista et al. (2020) [63] compared the qualities of handmade paper made from hemp pulp, eucalyptus pulp, and a combination of both fibers (100% hemp, 100% eucalyptus, and hemp/eucalyptus 50:50). The hemp plant had cooking yields that exceeded the wood, which is the reference. Moreover, the pulp showed better bleachability. In comparison to eucalyptus, hemp plants were able to produce pulp with a better gain in brightness and a smaller loss of intrinsic viscosity. The hemp pulp demonstrated improved breathability with a superior tearing resistance, and decreased air permeability. The paper sheets made from the hemp and eucalyptus mixture displayed intriguing features, which suggests a good pairing of these two raw ingredients for paper production. Danielewicz and Surma-Ślusarska (2017) [64] compared the papermaking from bleached kraft pulps derived from hemp stalks, woody-core, and bast fibers with that of bleached birch pulp and pine kraft pulp. Hemp stalk pulp has proven to give the best qualities for papermaking. Low tear resistance and low tensile strength were found in hemp woody-core pulps and hemp bast fiber pulp. Low internal fibrillation and low hemicellulose concentration of hemp bast fiber pulp are the main causes of its poor tensile strength. Mirski et al. (2017) [65] developed boards made of hemp fiber with densities ranging from 300 to 1100 kg/m^3^. Low swelling and low soaking susceptibility were two characteristics of hemp fiber boards that contributed to their comparatively strong water resistance. Fernea et al. (2017) [66] developed gypsum and hemp-based products. The findings indicate that the insulating materials had the best acoustic absorption and thermal conductivity as hemp volume increases. However, the smaller volume of hemp is advised to achieve high strength values. Kirilovs et al. (2020) [67] focused on an experimental analysis of the thermal conductivity and specific heat capacity of hemp fiber combined with phase change materials (PCMs). Direct incorporation of 5% encapsulate PCMs into the mass of hemp shives created the paperboards. The boards were made using cold pressing and Kleiberit urea formaldehyde resin glue as the adhesive. The heat capacity of the board is boosted by 62% by adding nanocapsules during the board production process. Danielewicz and Surma-Ślusarska (2019) [68] examined bleached kraft pulps made from mixtures of birch or pine with hemp stalks (80/20% weight). These pulps generally had similar characteristics to those of bleached pulps made from birch and pine. However, the air-resistance, and tearing resistance of birch or pine were significantly impacted when a hemp-woody core was substituted for a portion of the wood. Accordingly, hemp stalks are a superior fibrous raw material to hemp woody-core for papermaking. Kremensas et al. (2019) [69] investigated biocomposite boards made from various fiber fractions using hemp shivs as an aggregate and maize starch as a binder for thermal insulation. Various hemp shiv fractions have densities ranging from 81 to 96 kg/m^3^ and thermal conductivities between 0.0505 and 0.0616 W/(mK). The density of hemp shivs-based biocomposite boards was improved by 10 to 50% with the addition of maize starch, giving the lowest thermal conductivity ranges from 0.0605–0.0630 W/(mK).

#### 4.2.2. Textile

The varieties of hemp USO 31, Bialobrzeskie, Future 75, Carmagnola Selezionata, Santhica 27, and Santhica 70 originated from Europe are rich in high quality linen for further textile production [76]. The utilization of hemp in the apparel sector requires high-quality of fibers [24]. As a textile raw material, types of hemp fiber are divided into non-aligned short fiber and aligned long fiber [76]. The non-aligned fiber consists of a mixture that is chopped and developed when plant stems reach greater weight. Normally, non-aligned short fiber is used for non-woven fabrics. Meanwhile, aligned-long fiber is aligned during harvesting and eventual fiber extraction, utilizing as high-quality yarn spinning [76,77]. Long fiber yield reach one ton per hectare with variation among varieties. The study in Bottelare, Belgium indicated that long fiber yield ranged between 0.6–1.4 ton/hectare [76]. Recent utilizations of hemp in textile are shown in Table 3.

Grégoire et al. (2020) [85] reported that the fiber-rich hemp straw had a length of approximately 5 cm which is suitable for textile processing via carded route. Moreover, it has tensile strength and modulus elasticity up to 660 MPa and 38 GPa, respectively. These hemp straws are considered to be high enough for technical textile manufacturing such as load-bearing woven geotextile or composite reinforcements. Mechanical Kostic el al. (2008) [24] reported that slack modification improves tensile strength of hemp fiber up to 36.2 cN/tex compared to control (23.6 cN/tex).

Hemp fabric processing technology is similar to flax (linen) processing [76,86]. The fiber production steps include softening or lubricating, hackling/carding/drawing, spinning, weaving, and finishing/dyeing/bleaching [86]. The hemp spinning process can be both wet or dry, although, wet spinning will produce a finer yarn of hemp. Hemp fiber is also potent to be cottonized by removing the lignin content from 8–10% to 0.2%, resulting in finer, softer, and more workable textile in terms of cotton and wool systems, or even for blending with man-made fibers [86,87]. Similarly, it was also reported that hemp lignin treatment via enzymatic and alkali increased surface finesses and fiber flexibility [24]. Cotton-wool composited improved the amorphous phase, thus the flammability will be retarded [88,89].

As a raw material of textiles, hemp fiber has favorable characteristics: antimicrobial properties due to the presence of alkaloids, saponins, and flavonoids [90], high absorbency, good electrostatic properties, and low allergic risk. Moreover, hemp fabric is able to block UV up to 95% [88,89]. However, it has some drawbacks such as lack of cleanliness due to the presence of dust and shiv, fibers being too brittle and coarse, and high variability of fiber [24]. Thus, hemp fiber has a high potential to be a raw material in the fabric sector, therefore, high-quality of fiber is required.

#### 4.2.3. Composites and Plastic

The length and strength of hemp fiber make it ideal for composite/biocomposite production (Table 2 and Table 4), whereas hemp hurd, a byproduct of extracting bast fibers from the stalk, is used to make particle board and biodegradable plastics. A cradle-to-factory analysis of these composites showed that their incineration after use saved more energy and reduced greenhouse gas emissions as compared to their fossil-based counterparts. Polypropylene reinforced with hemp fiber are used in dashboards to replace synthetic polymers and copolymers such as polyacrylonitrile-butadiene-styrene as Yan et al. (2013) [50] developed hemp fiber and polypropylene composites by an internal mixing procedure. In this study, maleic anhydride modified polypropylene (MAPP) was included in composite materials containing 30% fiber. Tensile strength, flexural strength and impact strength increased from 28 MPa to 40 MPa, 42 MPa to 65 MPa and 22 to 33 kJ/m^2^, respectively. Beckermann et al. (2007) [48] compared composites containing untreated and alkali-treated hemp fiber. After combination in a twin-screw extruder, the composite materials including either treated or untreated fiber, polypropylene and MAPP coupling agent were injection molded into tensile test specimens. Composites containing alkali-treated fiber gave higher tensile strength than composites containing untreated fiber with addition of 4% MAPP. Luzi et al. (2016) [41] prepared PLA and PLA/PBS based films reinforced with unmodified and modified cellulose nanocrystals of hemp origin by solvent casting technique. Cellulose nanocrystal-PLA and -PLA 20PBS-based systems exhibited compact film structures with increased interfacial adhesion of the two polymeric phases. Dhakal et al. (2012) [51] used three different impactor geometries—hemispherical, conic with 30° and conic with 90° to test hemp fiber/unsaturated polyester composite laminates at four impact velocities: 2.52 m/s, 2.71 m/s, 2.89 m/s and 2.97 m/s. Results showed that specimens examined with hemispherical impactors withstood heavier weights, with maximum load greater than for impactor forms with 90° and 30°. Song et al. (2012) [91] developed hemp fiber/PLA composites of sandwich structure consisting of PLA sheets and hemp layers. Mechanical properties of these hemp fiber/PLA composites particularly the tensile strength and Young’s modulus increased at higher volume fractions. Pappu et al. (2019) [7] reported on hybrid fiber reinforced biodegradable composites by combining sisal and hemp fibers with polylactic acid, using melt processing and injection molding processes, while Zhang et al. (2020) [42] developed PVA and hemp fiber films by solvent casting. The hemp/PVA composite films showed more than a two-fold increase in Young’s modulus and tensile strength. Water vapor permeability of the pure PVA film was 0.98 × 10^−12^ g·cm/(cm^2^·s·Pa), while that of the composite films increased to a maximum of 1.14 × 10^−12^ g·cm/(cm^2^·s·Pa) when hemp content reached 10%. Sample performances under UV and visible light were compared at 280 nm to 500 nm wavelengths. Film with 10% hemp particles exhibited transmittance of 94.3% in visible light at 500 nm compared to 99.5% for pure PVA. Zhang et al. (2021) [43] prepared PVA and hemp fiber films by solvent casting. Hemp/PVA composite films showed increased Young’s modulus and tensile strength. Water vapor permeability increased and the film displayed UV-shielding properties when hemp fiber content increased. Wretfors et al. (2009) [52] reported on hemp fiber reinforced wheat gluten plastics. Tensile strength and Young’s modulus both rose considerably when short hemp fiber content increased. Mirpoor et al. (2022) [92] prepared protein-based films from hemp seed oilcake and observed that when enzymatically crosslinked proteins were used, tensile strength of hemp protein-based films increased, reaching a value of more than double the control films utilizing 20 U/g of microbial transglutaminase in protein pretreatment. Gironès et al. (2012) [39] developed starch-based composites reinforced with sisal and hemp fibers by melt processing. Tensile test results showed a continual increase in both tensile modulus and ultimate strength that was inversely correlated with reinforcement quantity. As a result, Young’s modulus for composites reinforced with 20% hemp strands showed values that were 18 times greater than non-reinforced composites.

Dixit et al. (2022) [99] produced novel alkali-treated hemp fiber/polyethylene/polypropylene composite films with optimal fraction for PE, PP and treated hemp fiber as 1.8, 1.118 and 1.2 g, respectively resulting in highest mechanical stability and lowest water vapor transmission rate. Previous studies found that addition of treated hemp fiber enhanced packaging properties such as contact angle, impact strength and transparency by 30%, 24% and 66%, respectively (with comparable mechanical and thermal stabilities to PE/PP composite film), while WVP decreased by 57% [99]. Mu et al. (2012) [100] utilized hemp haulm (by-product of hemp fiber extraction) as cushioning packaging materials that showed better cushioning properties than expanded polyethylene, and similar to expanded polystyrene cushions with applied stress above 5–15 N/cm^2^. Major factors affecting packaging properties were particle size, foaming agent and adhesives [100]. Barbash et al. (2022) [57] prepared nanocellulose from organosol (peracetic acid solution) hemp pulp (OHP) and found that addition of 2% hemp nanocellulose improved breaking force of the paper by 40% above the standard requirements for premium grade paper, while breaking length improved by 42%. These findings indicated that the use of hemp components effectively improved the properties of composite packaging materials as an alternative to conventional plastics. By creating composites with roughly 30 wt% fiber contents, Väisänen et al. (2018) [70] investigated potential of these fibers as reinforcements for epoxy resin. The findings indicate that, throughout a 28-day period, hemp fibers which undergone steam treatment absorbed the least water at 65% relative humidity (RH) and 20 °C, while enzymatic treatment produced the lowest water absorption values at 85% RH and 20 °C. Mechanical treatment of hemp fiber gave the highest tensile strength. The mechanical characteristics of epoxy-hemp composites were impaired by the alteration of hemp fibers, but water absorption values were dramatically reduced. Momeni et al. (2021) [71] created bio-based materials for packaging applications using poly(lactic acid) (PLA) biopolymer to enhance the value of hemp hurds. Effects of reinforcement content (5, 10, and 15 wt%) on mechanical and thermal characteristics of the biocomposites were determined. The biocomposites made from powdered treated hemp hurd showed improved thermal stability in the typical PLA processing temperature range of 130 to 180 °C. The Young’s modulus of the biocomposites made of PLA and containing 15% of hemp hurd powder was 2674 MPa, which is 9.3 percent higher than those of neat PLA and 70% higher than that of biocomposites made of PLA and containing the same weight percent of untreated hemp hurd powder. These findings reflected that hemp fibers had high potential to produce bio-based and biocomposite packaging. Enhanced properties can be achieved by several physical and chemical treatments.

## 5. Extraction and Composition of Hemp Extract

### 5.1. Solventless Extraction

Solventless extraction methods such as dry-sieving, water extraction and rosin press extraction are underrepresented in the literature. These simple procedures follow outdated techniques with difficulty in scaling. The finished hemp extract product can be further pressed into hashish or mixed with dried flowers. This straightforward procedure is time-consuming and labor-intensive, making it unsuitable for industrial use. Similar to dry sieving, this process is difficult to scale up and has limited potency control [101]. The common solventless method applied on cannabis are solid phase microextraction, dynamic/static head-space, and vacuum assisted sorbent extraction [102]. Static head-space technique is suitable for analysis of the volatile and residual solvent content of cannabis plant, which successfully eliminates the interference even from the heavy matrix. Solid phase microextraction is suitable for cannabis extraction for further application on edible and cosmetic industries as the process is natural. Dynamic headspace method technique use heating for a short equilibrium period and then the headspace above the cannabis sample is purged by high purity carrier gas and finally trapped in a sorbent-filled extraction tube.

### 5.2. Solvent-Based Extraction

Solvent-based extraction methods require a suitable liquid solvent such as ethanol, butane, propane, hexane, petroleum ether, methyl tertbutyl ether, diethyl ether, carbon dioxide (CO_2_) or olive oil [103,104,105,106], or gaseous solvents such as butane and propane [107]. Fiorito et al. (2022) [107] reported that the extraction of cannabinoids compound from hemp using n-butane relatively long at room temperature. The increasing temperature can shorten the extraction duration; however, it is may lessen of the chemical stability of extracted compound which also affected on overall yields. It was also found that n-butane effectively extracted cannabidiol, cannabichromene, trans-(8)-tetrahydrocannabinol, cannabidionic acid, and tetrahydrocannabinolic acid from hemp. Methods of extraction used include Soxhlet, static and dynamic maceration, ultrasonic-assisted extraction and microwave-assisted extraction.

#### 5.2.1. Soxhlet Extraction

Franz Ritter Von Soxhlet, a German scientist, first suggested the Soxhlet extraction technique, particularly for lipid extraction. This method gained popularity and is now frequently utilized for a variety of extraction tasks, most notably the separation of bioactive chemicals from plant matter. Soxhlet extraction is considered as a benchmark for evaluating and creating substitute separation techniques [108]. A small amount of dried material is first placed in a thimble and transferred to a distillation flask containing a specific solvent. A siphon is used to aspirate the solute for discharge into a distillation flask, with the extracted analyte carried along into the bulk liquid when the solution reaches overflow level. This method is performed multiple times until thorough extraction is complete [109]. Lewis-Bakker et al. (2019) [110] examined various types of organic solvents for cannabis extraction using the Soxhlet apparatus and found that ethanol provided optimal cannabinoid yields.

#### 5.2.2. Dynamic Maceration (DM)

Dynamic maceration is a common method for extracting solid lipids that involves soaking the sample in organic solvents (the solvent used depends on the polarity of the target component) for a predetermined time at a predetermined temperature and under stirring [111]. This affordable separation procedure is frequently used to acquire essential oils and beneficial substances [108]. Usage of vegetable oils (such as olive oil) as maceration extraction solvents was more effective than using alcoholic solvents for extracting larger concentrations of terpenes, especially at prolonged heating time. Vegetable oils are not volatile and challenging to remove from extracted isolates [105]. Another preferred solvent for cannabis extraction is ethanol. Fathordoobady et al. (2019) [111] used ethanol for neutral cannabinoid recovery, with no appreciable difference in results from other organic solvents (n-hexane, acetone or methanol). In the case of acidic cannabinoids the usage of ethanol as the solvent produced the highest yield. In cannabis maceration extraction, ethanol also generated maximum yield when employed twice compared to supercritical fluid extraction (SFE) or ultrasonic-assisted extraction (UAE) [108].

## 6. Application of Hemp Extracts in Packaging, Food and Textile

### 6.1. Polymeric Packaging

Hemp seed oil is a multipurpose crop with low environmental impact. Epoxidized vegetable oil (EVO)-based resins can be developed from hemp seed oil; a triglyceride with varying fatty acid compositions depending on plant, crop, season and growing conditions. Triglyceride units are formed by the esterification of a glycerol molecule and three fatty acid chains. Unsaturated fatty acid-containing vegetable oils are converted into reactive hydroxyl groups and used as natural polyols. Hemp oils are promising natural alternatives that show potential to increase shelf-life, microbiological safety and nutritional value of food. Shuttleworth et al. (2017) [9] developed a biocomposite by reacting cold-pressed hemp oil with peroxyacetic acid produced in situ to create flexible, transparent films with high water resistance, while Surender et al. (2016) [98] created a thermoplastic polyurethane that contained hydroxylated hemp seed oil, 1,10-methanediylbis(4-isocyanatobenzene), a linear polyester diol made from caprolactone monomer and a chain extender based on aromatic diol with a resorcinol base. The thermal stability and degradation mechanism changed as a result of hydroxylated hemp seed oil inclusion in the thermoplastic polyurethane structure. Cozmuta et al. (2015) [75] prepared hemp seed oil and gelatin film by solution casting. Film-forming solutions were examined for their wettability with various foods and their antibacterial effectiveness against *Escherichia coli, Staphylococcus aureus, Listeria innocua, Saccharomyces cerevisiae* and *Penicillium expansum*.

### 6.2. Food, Feed and Pharmaceutical Products

**Figure 5:**
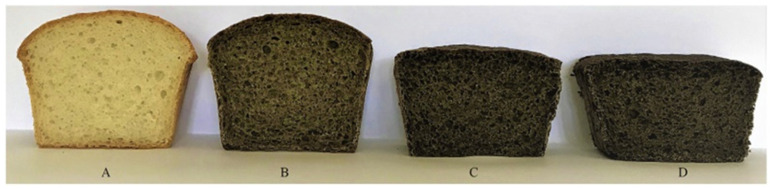
Transverse cuts of bread. Explanatory notes: (**A**)—wheat bread, (**B**)—bread with 15% hempseed flour, (**C**)—bread with 30% hemp seed flour, (**D**)—bread with 50% hemp seed flour (Reproduced with permission from Mikulec et al. (2019) [113]).

Hemp is a valuable addition to food [112]. Several applications of hemp in food, beverage and feed are shown in Table 5. Hemp seeds are a source of plant-based protein containing methionine, lysine and cysteine containing 20–25% protein, 25–35% lipids, 20–30% carbohydrates, 10–15% insoluble fibers, vitamins D and E and minerals such as phosphorus, potassium, sodium, magnesium, sulfur, calcium, iron and zinc [8,113,114,115,116,117]. Hemp flour increased the antioxidant properties of bread, with a darker color in bread products compared to the control (Figure 5) [113], and is also used to make gluten-free bread [118]. Using hemp flour or hemp seed cake reduced the volume of wheat bread [119,120].

Animal feed nutritional ingredients include hemp seed oil cake and meal. Hemp seed cake is effective as a protein supplement in animal feed due to its high crude protein content. Industrial hemp growers would benefit from the use of industrial hemp in commercial animal feed. Hemp seed oil meal has high fiber content, which limits its use, particularly in pig and poultry feed [128]. Linoleic acid and linolenic acid are the main omega-6 and omega-3 polyunsaturated fatty acids found in hemp seed oil [129]. Industrial hemp seeds are frequently used in bird seed mixes and as fish bait.

Pharmaceutical hemp contains bioactive compounds of human health interest [130], while hemp seed oil has been used as a medicine in China for at least 3000 years [131] to ameliorate stress, anxiety and pain as well as improve sleep and digestion. Hemp seed oil has also been used to treat cancer and cardiovascular disease, as well as to normalize cholesterol levels and blood pressure (Devi and Khanam 2019). Industrial hemp by-products, including inflorescences, represent a valuable resource for the pharmaceutical industry [132]. The use of hemp or marijuana as a drug has been regulated in some region by Colorado Code of Regulation (CCR), which focus on tetrahydrocannabinol (THC) as an active drug ingredient. THC content above 0.3% must be regulated by the Marijuana Enforcement Division in Colorado, while THC less than 0.3% has been excluded in medical interest and regulation [133].

## 7. Conclusions and Challenges

Hemp stalks, seeds and leaves can be used to make a variety of products including packaging, textiles, paper, food, beverages, cosmetics and pharmaceuticals. Hemp is a valuable economic crop with environmental benefits and high yield of natural products. Recent research has indicated several utilizations of hemp fiber as reinforcements in thermoplastic materials and paper. Physical and chemical modifications greatly enhanced the properties of hemp fibers by altering the fibrous structures (size and dimensions) with removal of impurities, chemical structure grafting and purifying the cellulosic components. Changes in the physical and chemical structures of hemp fiber improved the strength and thermal stability of composite polymeric materials. Findings support the utilization of novel sustainable bio-based materials to reduce the use of conventional petroleum-based plastics and additives. Hemp extracts have been used as edible food, animal feed and pharmaceutical applications to improve antioxidant and nutritional qualities. The extract has also been incorporated into active packaging to boost functionalized antimicrobial activity. High quality hemp fiber has been applied in apparel sector with several fiber modification. Hemp polymers and extracts are green materials with high potential for use in different industries including food and non-food applications. Poor barrier properties and compatibility in the composite materials are big challenge for hemp utilization in packaging industry. Further scaling up of fiber modification in pilot or commercial scale are necessary to ensure research utilization of hemp fiber in paper, textile and composite packaging. Moreover, regulatory concern and consumer perception of hemp extract as food ingredients are big challenge for applications as edible materials. These issues require more researches, regarding safety and health related issue in human and animal (for feed and pet food products). Further investigations are required to expand the potential of hemp in various industries and to create added value for the by-products.

## Figures and Tables

**Table 1:** Main components of hemp fiber.

Cellulose (%)	Hemicellulose (%)	Pectin (%)	Lignin (%)	Other (%)	Reference
67.0	16.1	0.8	3.3	2.8	[14]
74.4	17.9	0.9	3.7	0.8	[15]
74.0	18.0	1.0	4.0		[16]
55.0	16.0	18.0	4.0	7.0	[17]
76.0	11.5	1.3	3.2		[18]
57.0–77.0			9.0–13.0		[19]
75.1	<2.0		0.8		[20]
70.0–74.0	17.9–22.4	0.9	3.7–5.7	0.8	[21]
75.6	10.7		6.6		[22]
78.3			2.9		[23]
76.1	12.3	1.6	5.7	3.3	[24]

**Table 2:** Applications of hemp fiber in composite materials and paper.

Main Material	Type of Material	Application	Reference
Composite	Hemp fiber and epoxy resin	Reinforcements	[70]
	Hemp hurds and PLA	Reinforcements packaging	[71]
	Hemp hurd powder and PLA	Food packaging	[72]
	Hemp fiber and PLA	Reinforcements	[73]
	Hemp fiber and high-density polyethylene	Reinforcements	[74]
	Hemp fibers and polybenzoxazine	Green composite	[54]
	Hemp fibers and polybenzoxazine	Reinforcements	[55]
	Hemp hurd and polyvinyl alcohol solution	UV-shielding	[42]
	Hemp hurd and polyvinyl alcohol solution	UV-shielding	[43]
	Hemp seed oil and gelatin	Active packaging	[75]
Paper	Hemp fiber	Insulation material	[60]
	Root bast section of waste hemps	Air and oil filtration	[61]
	hemp stalks, PHA, PLA, PBS and PBSA	Food packaging	[62]
	Hemp pulp and eucalyptus pulp	Raw materials for papermaking	[63]
	Hemp pulp, birch pulp and pine pulps	Raw materials for papermaking	[64]
	Hemp fiber	Reinforced gypsum composite board	[66]
	Hemp shive	Phase change materials (PCMs)	[67]
	Hemp stalks, hemp-woody core, birch and pine	Raw materials for papermaking	[68]
	Hemp fiber and corn starch	Insulation material	[69]
	Hemp fiber	Furniture	[65]

**Table 3:** Related researches on application of hemp fiber in textile products.

Types/Additive/Composite Material	Processing Technology	Fabric Characteristic	Reference
Yellow colorant (*Buddleja officinalis*)	traditional techniques of dyeing from communities of Yunnan, China equipped with natural mordant treatment	Optimum dying achieved at pH 5, 60 °C for 90 min. Natural mordant treatment improved the yellowness and color fastness was maintained, indicating a good combination process between natural mordant treatment and natural yellow dyeing.	[78]
Graphited knitting hemp fabric (GKHF).	Furnace filled with a nitrogen atmosphere, heating at 800 °C	GKHF showed a great air permeability, water vapor and moisture, as well as remarkable static stability in the range of 0.5 to 480 KPa.GHKF detected a variety of static pressure and physiological signals for health monitoring, rehabilitation, and convenience sport stuff.	[79]
Feretiko hemp fiber (woven)	Thermal comfort evaluated by thermal manikin	Clothing insulation of Feretiko was 0.20, very close to ASHRAE standard 55-2013 for clothing insulation of a long-sleeve (0.25 clo). The air permeability was also high (2600 L/m^2^ s).	[80]
Hemp fabric and epoxy resin composite	Composite technology: plate and Impregnated Fiber Bundle Test (IFBT)	The pores of composite are low observed in tomography with the similar fiber volume for all the composite. Untwisting reduced the tenacity at break of the rovings. Low-twisted rovings of composite resulting in similar tensile strength of best flax in the range of 150–200 MPa.	[81]
Hemp fabric and vinyl ester composite	Chemical treated woven hemp fabric: NaOH and fire retardant (FR)	The treatment increase weight, thickness, density and yarn crimp, while decreased mechanical properties of woven fabric due elimination of hemicellulose and lignin by NaOH and hydrolyzation of cellulose by FR.The treatment increased thermal stability and limiting oxygen index values indicating fire retardant properties was improved.	[82]
Hemp fiber, Lyocell and PLA composite	Wrap spinning process	Lyocell addition improve tensile strength of hemp/PLA composite-based fabric and lesser fiber pull-outs appears, but did not affect on water absorption.	[83]
PLA	Compression molding technique	Reinforcement improved flexural and charpy detected for fibre volume fraction of 20 and 30%, and decreased at 40%. The impact strength increased by increasing reinforcement content. 30% reinforcement showed the best creep behavior	[84]

**Table 4:** Related researches on utilization of hemp components in packaging and composites.

Main Material	Packaging Technology	Material	Method of Hemp Fiber/Hemp Seed Oil	Packaging Properties	Reference
**Hemp fiber**	Internal mixerRoll mixerInjection molding machine	Hemp fiber and polypropylene	-	Tensile strength, flexural strength and impact strength increased from 28 MPa to 40 MPa, 42 MPa to 65 MPa and 22 to 33 kJ/m^2^, respectively.	[50]
	Twin-screw extruderInjection molding machine	Hemp fiber and polypropylene	Alkaline treatment	Tensile strength of composites containing alkali-treated and untreated fiber with addition of 4% MAPP increased by 50 and 40 MPa, respectively.	[48]
	Rapid-Kothen machine	Hemp fiber	Alkaline treatment	Hemp fiber paper had density of up to 1.56 g/cm^3^, tensile strength of up to 66.7 MPa and transparency of up to 87.3%	[57]
	Solvent casting	Hemp fiber, poly (lactic acid) (PLA) and poly (butylene succinate) (PBS)	-	Mechanical analysis of cellulose nanocrystal base film showed increased values of Young’s modulus but oxygen permeation decreased at 1.05-1.35 cm^3^ m^−1^ s^−1^ Pa^−1^.	[41]
	Compression molding process	Polyester and hemp fiber	-	Hemp fiber/unsaturated polyester composite laminates were subjected to impact testing with three distinct impactor geometries-hemispherical, conic with 30° and conic with 90° and four different impact velocities 2.52 m/s, 2.71 m/s, 2.89 m/s and 2.97 m/s. The findings of this study showed that specimens examined with a hemispherical impactor were able to withstand larger loads, with maximum load higher than for impactor forms with a 90° and 30° angle.	[51]
	Laminated in a sandwich-like structure	Poly (lactic acid)Hemp (*Cannabis sativa* L.)	-	Young’s modulus and tensile strength increased with increasing volume fraction.	[91]
	Twin-screw extruderInjection molding machine	Poly (lactic acid) and hemp fiber	-	Tensile strength (46.25 ± 6.75 MPa), Young’s modulus (6.1 ± 0.58 GPa), flexural strength (94.83 ± 11.21 MPa), flexural modulus (6.04 ± 0.55 GPA), density (1.14 ± 0.07 g/cm^3^), elongation at break (0.93 ± 0.35%) and water absorption capacity (1.06 ± 0.18%) of hybrid fiber composites improved compared to neat PLA.	[7]
	Single-screw extruder	Potato starch and hemp fiber	-	Elastic modulus showed that the interphase effect changed favorably as fiber content increased.	[93]
	Compression	Hemp fiber and polyethylene	-	Hemp fiber composite had modulus of elasticity and rupture, and flexural strain at break 8.0 ± 0.4 GPa, 110.8 ± 5.0 MPa and 4.2 ± 0.3%, respectively. Tensile modulus, strength and elongation at break were 4.1 ± 0.2 GPa, 67.6 ± 0.2 MPa and 3.5 ± 0.3%, respectively.	[94]
	Solvent casting	Hemp hurd and polyvinyl alcohol solution (PVA)	Steam explosion treatment	Young’s modulus and tensile strength of hemp/PVA composite films increased more than two-fold. When hemp fiber content increased, water vapor permeability increased and film was UV-shielding.	[42]
	Laminates	Hemp fibers or flax fibers and epoxy resins (EP) or polypropylene (PP)	Alkaline treatment	Fiber characteristics caused by mercerization increased flexural modulus and flexural strength of composites by 100% and 45%, respectively.	[95]
	Solvent casting	Hemp hurd and polyvinyl alcohol solution (PVA)	-	Young’s modulus and tensile strength increased more than two-fold in hemp/PVA composite films. When hemp fiber content increased, water vapor permeability increased and film was UV-shielding.	[43]
	Compression molding	Hemp fiberWheat gluten	-	Tensile strength and Young’s modulus both dramatically increased with increasing short hemp fiber percentage.	[52]
	Solvent casting	Hemp (*Cannabis sativa* L.) seed oilcake	-	Tensile strength of hemp protein-based films increased when enzymatically crosslinked proteins were used, reaching more than double the control films using 20 U/g of mTGase in the protein pretreatment.	[92]
	Compression molding	Hemp fibers and cashew nut shell liquid matrix	Alkaline treatment	The 4 and 6% NaOH treatments gave the maximum Young’s modulus and tensile strength of 65 GPa and 1064 MPa, respectively when fibers were tested in tension.	[96]
	Compression molding	Hemp fibers and polybenzoxazine	Alkaline treatment	Tensile strength and Young’s modulus values increased as waste hemp fibers loading increased.	[55]
	Melt processing	Hemp fibers and corn starch	-	According to DMTA analysis, hemp fibers increased glass transition temperature (Tg) of TPS. Reinforcement also enhanced material stiffness, reflected in storage modulus and Young’s modulus values.	[39]
	Resin transfer molding	Hemp fiber and unsaturated polyester	-	Increasing fiber content linearly increased material tensile, flexural and impact capabilities.	[97]
	Compression molding	Hemp fibers and polybenzoxazine	Alkaline and silane treatment	Silane-treated fiber composites demonstrated optimal properties compared to other treated fiber composites for flexural, tensile and impact tests.	[54]
	Hydraulic hot-press	Hemp fibers and polybenzoxazine	Alkaline treatment	Impact, tensile strength, flexural and Young’s modulus increased after loading waste hemp fibers.	[53]
**Hemp seed oil**	Planetary centrifugal mixer	4-dimethylaminopyridine (DMAP) and hemp seed oil	Cold-pressed hemp oil	This biocomposite was used to form flexible and transparent films with high water resistance.	[9]
	Hydrolyzed hemp seed oil	Hemp seed oil	Cold-pressed hemp oil	Thermal stabilities and degradation mechanisms were altered by adding hydroxylated hemp seed oil which changed the thermoplastic polyurethane structure.	[98]
	Film-forming solutions	Hemp seed oil and gelatin	Cold-pressed hemp oil	Film-forming solution had a significant additive inhibitory impact against *Staphylococcus aureus*, *Listeria innocua*, *Saccharomyces cerevisiae* and *Penicillium expansum* and a moderate additive inhibitory effect against *E. coli*.	[75]

**Table 5:** Related researches on applications of hemp in food and feed.

Product Type	Product Form	Mixed Ingredient	Technology	Observation Result	Reference
Food	Wheat bread	Hemp and heat flour	single-phase method in a fast rotating spiral mixer	Wheat bread containing hemp flour had higher protein content (13.38–19.29 g/100 g d.m) compared to white bread (11.02 g/100 g d.m), but reduce sensory characteristic. Moreover, bread stalling is reduced indicating hardness changing inhibition, increased browning index from 29.69 to 46.26 and phenolic content.	[113]
	Gluten free bread	Hemp flour, corn starch, potato starch,	Baking using convection oven	Hemp flour weakened starch-based gluten free bread structure, while 20% reinforced the structure. Hemp flour improved dietary fiber content and prevent the hardening, but reduced the lightness.	[118]
	Bread	Hemp and wheat flour	Baking	Hemp flour improved the shelf-life of wheat bread, while reduced dough consistency up to 82%.	[121]
	Sponge cake	Hemp, pea, and insect protein	Baking using electric oven	Combination of 3.75% pea, 3.75% hemp, and 7.5% insect was possible to obtain egg-free sponge cake.	[122]
Beverage	Fermented plant-based drink		Fermentation: *Lactobacillus fermentum, Lb. plantarum, and Bifidobacterium bifidum*.	Hemp seed based probiotic drink showed a strong prebiotic activity and bioactive compound improvementAcetate, propionate, and butyrate contained in hemp seed functioned to select the growth of beneficial microbes.	[123]
Feed	Dietary hemp seed diet rich in ω-6 polyunsaturated fatty acid (PUFA) for sows			Hemp diet positively influence the activities of antioxidant enzymes and nitric oxide production level in sows plasma, indicating the reducing of lipid oxidation. It improved antioxidant status of lactating sows and their progeny.	[124]
	Hemp oil for pig			The hemp oil improved alpha linoleic acid in the pork	[125]
	Hemp oil and hemp omega for chicken broiler and laying hens			Chicken broiler and laying hens feed with hemp oil and hemp omega had greather total n-3 polyunsaturated fatty acid	[126]
	Hemp seed for alpine goats			Hemp seed increased the iron content in alpine goat blood from 33 to 67%, confirmed by high phytate content in hemp seed.	[127]

## Data Availability

The data presented in this study are available on request from the corresponding author.
